# A New Method of Litter Equalization in Rabbit

**DOI:** 10.3390/ani15111644

**Published:** 2025-06-03

**Authors:** Tamás Atkári, Zsolt Gerencsér, István Nagy, Zsolt Szendrő

**Affiliations:** 1 Olivia Ltd., Mizse 94, 6050 Lajosmizse, Hungary; tamas.atkari@olivia.hu; 2 Institute of Animal Sciences, Kaposvár Campus, Hungarian University of Agriculture and Life Sciences, Guba Sándor Str. 40, 7400 Kaposvár, Hungary; nagy.istvan.prof@uni-mate.hu; 3 Institute of Physiology and Nutrition, Kaposvár Campus, Hungarian University of Agriculture and Life Sciences, Guba Sándor Str. 40, 7400 Kaposvár, Hungary; szendro.zsolt@uni-mate.hu

**Keywords:** rabbit, litter homogenization, body weight, carcass

## Abstract

On rabbit farms, the mortality of rabbits born with a low weight is high, and there is a large variation in body weight, so the carcass and the cut carcass are not uniform. The experiment aimed to reduce the mortality of kits born with a low weight and to produce more-uniform slaughter animals. In the experiment, each rabbit doe in the Control group raised 10 newborn kits with low birth weights (S10, *n* = 100), 10 with medium birth weights (M10C, *n* = 100), or 10 with high birth weights (L10, *n* = 100). In the Experimental group, the rabbit does raised 9 newborn kits with low birth weights (S9, *n* = 90), 10 with medium birth weights (M10E, *n* = 100), or 11 with high birth weights (L11, *n* = 110). Compared with the S10 group, the mortality (between 0 and 7 days) in the S9 group was reduced (20.0% vs. 8.9%; *p* < 0.001). While the body weights of the S10, M10C, and L10 subgroups in the Control group increased at 84 days (2876 g, 2872 g, and 3047 g, respectively), there was no significant difference in the body weights of the three subgroups (S9, M10E, and L11) in the Experimental group. The new litter-equalization method was therefore suitable for reducing mortality and achieving a more-equal slaughter weight. At the same time, there was no difference between the groups in the dressing out percentage. The authors further suggest considering the teat number of the does and raising low-weight kits in litters of eight.

## Simple Summary

In order to achieve uniform appearance of the carcass and parts of the carcasses, it would be optimal if all rabbits weighed the same at slaughter. However, fattening lasts until a certain age, i.e., 11 weeks, at which time the rabbits’ body weight varies greatly. It is desirable to reduce this variation. In practice, the mortality of rabbits born with a low weight is reduced by placing kits of similar birth weight in one litter, forming separate litter groups of kits having low, medium, and high birth weights, respectively. In the experiment, the authors investigated the advantage of placing 9 low-weight, 10 medium-weight, and 11 large newborn rabbits in a litter. According to the results, fewer rabbits born with a low weight died, but the slaughter weight homogeneity of the rabbits improved only slightly.

## 1. Introduction

Over a vast number of years, the rabbit sector has made significant contributions to rural economic growth and social development in many countries. This progress is largely due to the industry’s favorable features, including low investment requirements, ease of commencement and management, efficient use of grain and terrestrial resources, and the production of superior meat [1].

It is advantageous from several aspects (space requirements, feed requirements, etc.) if rabbits of the same age have similar body weights. The most important issue is that the weight is balanced at slaughter time, because it is an important aspect of automating the slaughter, and the packaged product (carcass, backbone, thigh, etc.) is then uniform [2,3,4]. However, several factors influence body weight during fetal life and until weaning.

Many factors influence the growth of fetuses. The uterus of heavier female rabbits is larger and has more space for the fetuses [5,6]. The primiparous female rabbit is not yet fully developed [7], but birth weight may increase even up to the fourth to sixth litter [8]. One of the most significant effects is litter size. In larger litters, the birth weight decreases, but the standard deviation of the birth weight increases [9,10]. This is because, as the number of fetuses increases, their weight decreases, but the position of the fetuses within the uterus also affects their weight. The largest fetuses are found at the end of the uterus facing the ovaries, and fetal weight decreases toward the cervix, with foetuses of average weight found near the cervix [11,12,13]. The weight of the fetuses is decisively influenced by their nutrient supply, i.e., how many blood vessels connect them with the maternal placenta; the more blood vessels, the greater their weight [13,14,15]. The reproductive rhythm, the extent to which lactation and the next gestation overlap, and when the rabbits are weaned, all affect the growth of the fetuses, as the feed (energy) consumed by the rabbit does is divided between lactation and gestation in addition to life requirements [16,17,18].

The selection of intrauterine conditions that were favorable for within-litter homogeneity of the birth weight proved effective [19,20,21]. However, several factors during the lactation period can increase the variation in the weight of rabbits.

The effect of litter size is also significant after birth, as more or fewer kits share the available milk. Kits born with lower birth weights, especially in large litters, are disadvantaged in the competition for milk. A correlation has been found between birth weight and milk consumption [22]. Other studies have also found differences in milk consumption between kits born with small, medium, and large birth weights [23,24]. The disadvantage of kits with low birth weights is reflected not only in lower weight gain but also in higher mortality [25,26].

Heavier kits have an additional advantage by moving into the middle, warmer part of the nest, while smaller ones are placed at the edge of the nest, where they are only warmed by their littermates from one side [27,28]. Smaller rabbits may lose their brown fat tissue, which is used to warm their bodies in the cold [29,30], further increasing their chances of being cold.

Some researchers have examined the effect of the number of rabbits in a litter and the birth weight [31,32]. Litter equalization was carried out on rabbit farms to ensure that kits had a similar chance of survival and proper growth. Initially, only the same number of rabbits were placed in each litter, but later the rabbits’ weight within the litter was similar [32]. Usually, 8 rabbits were placed under primiparous mothers, and 10 rabbits under multiparous mothers.

The aim of this study is twofold. On the one hand, it aims to improve the survival rate of kits born with a low birth weight, and on the other hand, it aims to reduce the variation in body weight and carcass weight of rabbits by placing fewer kits born with a low birth weight and more kits born with a high birth weight in a litter.

## 2. Materials and Methods

This investigation did not require special permission. All the animals were handled according to the principles outlined in the EC Directive 2010/63/EU on protecting animals used for scientific and experimental purposes. Since the rabbits were housed and slaughtered according to the regular protocols at the slaughterhouse of Olivia Ltd. (Lajosmizse, Hungary) no special permit was required to experiment in Hungary.

### 2.1. Animals, Housing, Feeding

The experiment was conducted from February 2020 to May 2021 at the Oligen Farm (Olivia Ltd.; 47°13′57″ N 19°26′00″ E). The rabbit does and their kits were housed in Landkaninchen technology. It had a floor area of 81 × 53 cm, an elevated platform of 42 × 53 cm, and a nest box of 22 × 53 cm; its height was 60 cm, and it was equipped with a hay pocket. After weaning, the rabbits were placed in a cage with a 43.5 × 23.4 cm floor area (2 rabbits/cage) (Meneghin Rendita series 8 1.5P, Meneghin Ltd., Povegliano, Italy).

At the time of kindling, the temperature in the rabbit house was 18 °C, which was later reduced to 15 °C. A 12 h daily lighting program was used, which was increased to 16 h in the week before artificial insemination for biostimulation purposes and then gradually reduced to 12 h again. During fattening, the temperature was 18 °C, the humidity was 40–60%, and the daily lighting was 12 h. Although an artificial lighting program was used, the buildings were equipped with windows to let in external light.

The animals received commercial pellets ad libitum. The chemical composition of the pellets was as follows: does and their kits—crude protein (CP): 18.1%, crude fiber (CF): 15.3%, digestible energy (DE): 11.12 MJ/kg; after weaning (at 36 days of age)—CP: 18%, CF: 17.8%, DE: 10.36 MJ/kg; at the end of the fattening period—CP: 17.1%, CF: 15.9%, DE: 10.41 MJ/kg. Drinking water was available ad libitum from nipple drinkers. Antibiotic treatment was not applied.

### 2.2. Experimental Groups

**Figure 1:**
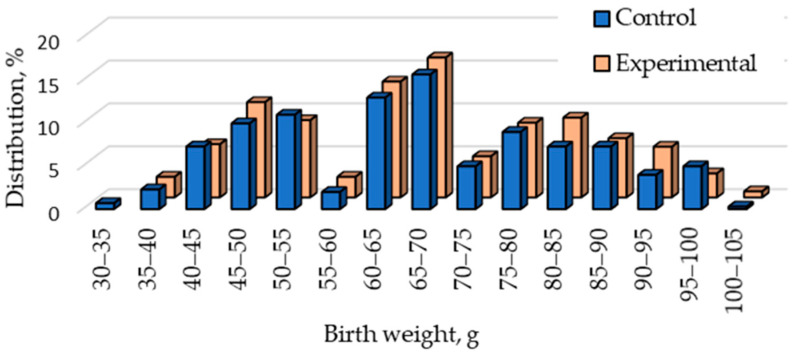
Distribution of birth weight in the Control group (Control: 10 small, 10 medium, or 10 large) and the Experimental group (Experimental: 9 small, 10 medium, or 11 large).

The experiment was conducted in two replicates with Pannon White rabbits. The origin and selection of this medium-sized meat rabbit breed have been described in detail previously [33]. This breed’s average birth weight and its distribution correspond to those of other medium-sized breeds [34]. Multiparous does that did not show any reproductive problem were selected. At birth, their kits were removed, and, under the experimental design, small (35–55 g), medium (60–70 g), and large (75–100 g) newborn rabbits were given in equal proportions (*p* = 0.745). Two groups were formed. In the first group (Control), 10 small (S10, *n* = 100), 10 medium (M10C, *n* = 100), or 10 large (L10, *n* = 100) newborn rabbits were placed with each doe in accordance with usual farm practice. In the other group (Experimental), 9 small (S9, *n* = 90), 10 medium (M10E, *n* = 100), or 11 large (L11, *n* = 110) newborn rabbits were placed with each doe. The formation of groups was not a problem, because litter size equalization with cross-fostering is a commonly used method on rabbit farms [35] to reduce mortality [36], and rabbit does readily accept the offspring of other mothers. The kits were placed in mixed-sex litters because sexual dimorphism in rabbits is thought to either not exist or to arise only late in life [37]. Figure 1 shows the distribution of newborn rabbits in the two groups according to their body weight.

### 2.3. Measurement

The kits were individually marked with newborn ear tags (Simplex Baby, Chevillot, France), and their weight was measured weekly until they reached 12 weeks of age. The rabbits were measured at each Monday tie-up. The rabbits were measured with a digital scale (Bizerba HW-E16500W, Balingen, Baden-Württemberg, Germany). To prevent injury and avoid movement during the measurement, the rabbits were placed in a small box. The rabbits were measured by a veterinarian (Dr. Tamás Atkári), who has over 20 years of experience caring for rabbits without causing injury to the rabbits or the caretaker. Their daily weight gain was calculated using these measurements. The calculation method was as follows: average daily gain = (final weight − initial weight)/number of days between the two measurements. The number of dead rabbits was recorded daily.

After 12 weeks of age, the slaughtering of rabbits took place at the slaughterhouse of Olivia Kft in Lajosmizse. The rabbits were delivered to the slaughterhouse in a standard plastic transport container used in the rabbit industry. The transport duration was 1 h (the distance was 35 km), and they were transported 2 h before the slaughter. During the transport period (from loading to actual slaughter), the rabbits lose weight as they no longer have food available. This loss depends on the animal’s physiological status but can be between 50 and 150 g. Regarding transport, we maintained the national welfare control standards. After a short resting period, the rabbits were bled following electric stunning. The skin, viscera, etc. were removed according to the slaughterhouse protocol. In the final phase of slaughter, the weights of the carcasses were measured, and the dressing out percentage was calculated in relation to the body weight before slaughter according to Blasco and Ouhayoun (1996) [38].

### 2.4. Statistical Analysis

The online version of the SAS software package (3.81, Enterprise Edition) was used for statistical analysis. The body weights measured weekly were analyzed using a Generalized Linear Mixed model, taking into account the repeated measurements. The rabbits and their does were defined as random effects in which levels of rabbits were nested within levels of does. Differences among the groups by each week were determined using the SLICE statement. The slaughter weight and the carcass traits were evaluated by One-Way ANOVA, while the mortality data and the homogeneity of the birth and 12 weeks weights’ frequency distributions between the Control and Experimental groups were evaluated by the Chi^2^-test.

## 3. Results and Discussion

### 3.1. Growth Traits

The rabbits’ body weight was significantly higher in the control group from the age of 28 days than that in the Experimental group (Table 1). However, at 84 days, the 51 g difference between the two groups was practically negligible.

**Figure 2:**
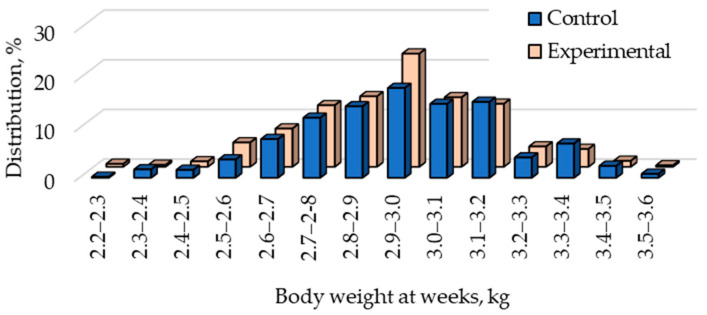
Distribution of body weight at 12 weeks in the Control group (Control: 10 small, 10 medium, or 10 large) and the Experimental group (Experimental: 9 small, 10 medium, or 11 large).

There was also a non-significant difference (*p* = 0.106) in the distribution of body weight at 12 weeks of age between the two groups (Figure 2).

We expected a larger difference between the standard deviations (heterogeneity) of the two groups. Table 2 explains the reason for this. It was expected that the body weight in the S9 group would be greater than that in the S10 group because one less rabbit was placed in the S9 litters. Consequently, they receive more milk per kit [39] and therefore gain more weight during the weaning period. However, the difference between litter size in the S10 and S9 groups changed due to mortality (Table 3).

The M10C and M10E groups were identical (Table 2); in both cases, there were 10 medium-weight rabbits in a litter in both groups. Therefore, despite some random variations, there was no significant difference in their body weights. At the same time, significant differences emerged between the L10 and L11 groups by day 14, which continued to the end of the experiment (Table 2). Since 0–1% of kits from these groups died between birth and 7 days of age (Table 3), the amount of milk per rabbit was lower due to the larger number of rabbits per litter sharing it. Figure 2 also shows fewer rabbits in the largest weight categories from the Experimental group than from the Control group. This and the lower average weight of the L11 group explain why the SD in the Experimental group was slightly lower than that in the Control group.

Without litter equalization, when kits between 39 and 70 g were placed in litters of 6, 8, and 10, and in the case of litter equalization, when only kits with low, medium, or high birth weight were nursed by the doe [31], the equalization slightly increased the 10-week body weight for all litter sizes and all weight categories, which was significant for birth weights of 39–43 g. In addition, the variance (SE) of body weight also decreased significantly. In that study [31], there was a greater difference between litter sizes, and there were two kits less in each litter than in the current experiment, but the results demonstrate that litter equalization reduces the variance of body weight. Regardless of litter size, small rabbits reached a weight similar to that of those raised in litters of 10, regardless of their birth weight; medium-weight rabbits reached a weight similar to that of those nursed in litters of 8; and large newborns reached a weight similar to that of those nursed in litters of 6. In another experiment, the rabbits were examined before weaning [32]. Newborn rabbits in litters of six consumed more milk and therefore grew better than those raised in litters of eight. Despite this, due to the higher initial weight and appetite, the rabbits raised in litters of eight had the highest body weight at 35 days of age. These results show that the effect of litter size and birth weight is most significant during the lactation period, with compensatory growth rarely observed after weaning.

### 3.2. Mortality

More than twice as many kits died in the first week in the Control group as in the Experimental group (Table 3). This is because small kits born with a low weight are less viable than larger, heavier ones, and there was also a clear effect caused by the number of kits nursed by the doe [32]. Although there was a one kit difference between the S10 and S9 groups, this difference was almost equalized due to the higher mortality rate in the S10 group. Thus, in the end, rabbits in both groups could access a similar amount of milk, and therefore their body weight also developed similarly; there was no significant difference in their weights at 12 weeks.

A low birth weight and a large litter size had a negative impact on mortality. The beneficial effect of litter equalization on mortality has been demonstrated [31]. In one experiment, litters of 6, 8, and 10 were created, each containing kits weighing between 39 and 70 g. In another experiment, three groups were created based on the birth weight, and only low-weight, medium-weight, or heavy-weight rabbits were placed in them. Due to litter equalization, mortality in the first 3 weeks of life in litters of 10 was almost halved (22.2 vs. 12.0%), and mortality in small- and medium-weight kits was also significantly reduced. Other authors have reported results similar to those of the present experiment [31]. Regardless of the litter size (6, 8, 10), mortality among low-birth-weight kits was similar (about 20%) to that when the rabbit does nursed 10 kits regardless of birth weight (40–49, 50–59, 60–69 g). Six or eight kits weighing 45 g and eight kits weighing 70 g were placed in a litter [32]. If the does nursed eight low-birth-weight kits, the mortality was higher than that in the other two groups.

The number of teats may have played a role in the mortality of rabbits born with a low birth weight [40] since weak individuals only have a chance to access teats and a sufficient amount of milk if the number of teats is not less than the number of rabbits in the litter [41].

### 3.3. Carcass Traits

Similarly to the case for body weight at 84 days of age, there was no significant difference between carcass weights in groups S10 and S9, and M10C and M10E; however, the carcass weight of L10 rabbits was 84 g higher than that of L11 rabbits (Table 4). As a result, the carcass weights of S9, M10E, and L11 rabbits in the Experimental group were similar, which means that they became more balanced, in accordance with the experimental objective. There was no significant difference in slaughter weight between the Experimental and Control groups nor between the groups based on birth weight (Table 4). The standard deviation in the Experimental group decreased slightly (139 vs. 132 g).

We have not found any literature source that examines the effect of birth weight and litter size on slaughter traits; therefore, there is no basis for comparing our results.

## 4. Conclusions

In the present experiment, placing only 9 small- and 11 large-birth-weight newborn kits in a litter instead of 10 was advantageous. The mortality of suckling kits and the variability of body weight at slaughter and carcass weight can be reduced.

Further studies are needed to examine the combined effect of birth weight and teat number on kit mortality, to reduce the variance in body and carcass weight, and to raise low-weight kits in litters of eight.

## Figures and Tables

**Table 1:** Body weight of rabbits (g) between birth and 12 weeks of age in the Control and Experimental groups.

Age, Days	Control Group		Experimental Group	
	Lsmean	SD	Lsmean	SD
*n*	300	-	300	-
At birth	66.3	16.6	66.8	16.3
7	160	29.6	158	26.8
14	280	41.1	269	40.9
21	425	55.1	404	59.5
28	682 ^b^	82.7	654 ^a^	86.8
35	1098 ^b^	112	1062 ^a^	125
42	1348 ^b^	168	1324 ^a^	173
49	1645 ^b^	154	1596 ^a^	153
56	1928 ^b^	168	1881 ^a^	158
63	2183 ^b^	187	2139 ^a^	166
70	2430 ^b^	201	2382 ^a^	179
77	2698 ^b^	226	2648 ^a^	212
84	2971 ^b^	238	2921 ^a^	219

Note: Control group = Only small, medium, or large newborn rabbits in a litter; Experimental group = Fewer small rabbits, more large rabbits in a litter. ^a,b^ Different superscripts in the same row indicate significant differences (*p* < 0.05).

**Table 2:** Body weight of rabbits (g) born with small, medium, and large weights between birth and 12 weeks of age in the Control and Experimental groups.

Age, Days	Control Group			Experimental Group			RMSE	Prob
	S10	M10C	L10	S9	M10E	L11		
*n*	100	100	100	90	100	110	-	-
At birth	47.0 ^a^	65.9 ^c^	85.2 ^d^	47.4 ^ab^	65.0 ^bc^	85.1 ^d^	5.57	<0.001
7	134 ^a^	165 ^b^	178 ^b^	133 ^a^	169 ^b^	169 ^b^	22.7	<0.001
14	258 ^ab^	285 ^cd^	294 ^d^	249 ^a^	288 ^d^	269 ^bc^	38.5	<0.001
21	406 ^b^	430 ^c^	438 ^c^	405 ^b^	428 ^c^	385 ^a^	55.6	<0.001
28	641 ^a^	683 ^b^	715 ^c^	633 ^a^	690 ^b^	641 ^a^	80.6	<0.001
35	1043 ^a^	1103 ^b^	1137 ^c^	1043 ^a^	1099 ^b^	1045 ^a^	115	<0.001
42	1290 ^a^	1359 ^cd^	1386 ^d^	1302 ^ab^	1355 ^bcd^	1314 ^abc^	168	<0.001
49	1595 ^ab^	1640 ^bc^	1690 ^c^	1574 ^a^	1627 ^ab^	1588 ^a^	151	<0.001
56	1874 ^ab^	1926 ^bc^	1975 ^c^	1853 ^a^	1906 ^ab^	1884 ^ab^	160	<0.001
63	2125 ^ab^	2178 ^b^	2235 ^c^	2105 ^a^	2162 ^b^	2149 ^ab^	174	<0.001
70	2356 ^a^	2423 ^b^	2498 ^c^	2357 ^a^	2398 ^ab^	2390 ^ab^	186	<0.001
77	2617 ^a^	2693 ^b^	2771 ^c^	2623 ^a^	2669 ^ab^	2656 ^ab^	215	<0.001
84	2876 ^a^	2972 ^c^	3047 ^d^	2893 ^ab^	2933 ^abc^	2936 ^bc^	223	<0.001

Note: S10 = 10 small; M10C = 10 medium, control; L10 = 10 large; S9 = 9 small; M10E = 10 medium, experimental; L11 = 11 large. ^a–d^ Different superscripts in the same row indicate significant differences (*p* < 0.05).

**Table 3:** Mortality of rabbits (%) between birth and 12 weeks of age in the Control and Experimental groups.

Age, Days	Control Group			Experimental Group			Prob
	S10	M10C	L10	S9	M10E	L11	
*n* (at birth)	100	100	100	90	100	110	-
0–7	20.0 ^c^	1.0 ^a^	1.0 ^a^	8.9 ^b^	1.0 ^a^	0.0 ^a^	<0.001
0–35	22.0 ^c^	9.0 ^b^	2.0 ^a^	11.1 ^b^	10.0 ^b^	0.9 ^a^	<0.001
*n* (at weaning)	78	91	98	80	90	109	
35–84	5.1	2.2	6.1	2.5	4.4	1.8	0.518

Note: Control group = Only small, medium, or large newborn rabbits in a litter; Experimental group = Fewer small rabbits, more large rabbits in a litter. ^a–c^ Different superscripts in the same row indicate significant differences (*p* < 0.05).

**Table 4:** Carcass traits of rabbits born with small, medium, and large weights between birth and 12 weeks of age in the Control and Experimental groups.

Carcass Traits	Control Group			Experimental Group			RMSE	Prob
	S10	M10C	L10	S9	M10E	L11		
*n*	40	79	46	37	79	52	-	-
Body weight at slaughter, g	2800 ^a^	2920 ^ab^	3001 ^b^	2884 ^ab^	2841 ^a^	2843 ^a^	222	0.001
Weight of carcass, g	1655 ^a^	1689 ^a^	1747 ^b^	1656 ^a^	1663 ^a^	1662 ^a^	133	<0.001
Dressing out percentage, %	59.0	59.1	59.0	58.8	58.7	59.5	1.57	0.188

Note: S10 = 10 small; M10C = 10 medium, control; L10 = 10 large; S9 = 9 small; M10E = 10 medium, experimental; L11 = 11 large. ^a,b^ Different superscripts in the same row indicate significant differences (*p* < 0.05).

## Data Availability

The original contributions presented in this study are included in the article.
